# Screening for Atrial Fibrillation: Improving Efficiency of Manual Review of Handheld Electrocardiograms ^†^

**DOI:** 10.3390/ecsa-7-08195

**Published:** 2020-11-14

**Authors:** Madhumitha Pandiaraja, James Brimicombe, Martin Cowie, Andrew Dymond, Hannah Clair Lindén, Gregory Y. H. Lip, Jonathan Mant, Kate Williams, Peter H. Charlton

**Affiliations:** 1 Primary Care Unit, Department of Public Health and Primary Care, University of Cambridge, Cambridge CB1 8RN, UK; 2 Faculty of Medicine, National Heart & Lung Institute, Imperial College London, London SW3 6LY, UK; 3 Zenicor Medical Systems AB, 113 59 Stockholm, Sweden; 4 Liverpool Centre for Cardiovascular Science, University of Liverpool and Liverpool Heart and Chest Hospital, Liverpool L69 7TX, UK

**Keywords:** algorithms, electrocardiography, atrial fibrillation, screening, handheld sensors

## Abstract

Atrial fibrillation (AF) is a common irregular heart rhythm associated with a five-fold increase in stroke risk. It is often not recognised as it can occur intermittently and without symptoms. A promising approach to detect AF is to use a handheld electrocardiogram (ECG) sensor for screening. However, the ECG recordings must be manually reviewed, which is time-consuming and costly. Our aims were to: (i) evaluate the manual review workload; and (ii) evaluate strategies to reduce the workload. In total, 2141 older adults were asked to record their ECG four times per day for 1–4 weeks in the SAFER (Screening for Atrial Fibrillation with ECG to Reduce stroke) Feasibility Study, producing 162,515 recordings. Patients with AF were identified by: (i) an algorithm classifying recordings based on signal quality (high or low) and heart rhythm; (ii) a nurse reviewing recordings to correct algorithm misclassifications; and (iii) two cardiologists independently reviewing recordings from patients with any evidence of rhythm abnormality. It was estimated that 30,165 reviews were required (20,155 by the nurse, and 5005 by each cardiologist). The total number of reviews could be reduced to 24,561 if low-quality recordings were excluded from review; 18,573 by only reviewing ECGs falling under certain pathological classifications; and 18,144 by only reviewing ECGs displaying an irregularly irregular rhythm for the entire recording. The number of AF patients identified would not fall considerably: from 54 to 54, 54 and 53, respectively. In conclusion, simple approaches may help feasibly reduce the manual workload by 38.4% whilst still identifying the same number of patients with undiagnosed, clinically relevant AF.

## 1 Introduction

Atrial fibrillation (AF) is the most common cardiac arrhythmia globally and is thought to affect approximately 3.3% of the UK population [1]. AF confers a five-fold increase in stroke risk [2], and is associated with approximately 28% of all strokes [3]. If AF is diagnosed, the associated risk of stroke can be reduced by approximately 60% through anticoagulation [4]. However, AF can be asymptomatic and can occur only intermittently, making it difficult to diagnose. Consequently, approximately three in ten cases of AF are not diagnosed [5]. With the global prevalence of AF expected to more than double by 2050 [6], screening for AF is being explored as a strategy to tackle this growing public health issue.

Screening for AF holds promise for identifying undiagnosed AF, even if it only occurs intermittently and asymptomatically. Screening is typically targeted at older adults in whom most cases of AF are found [5]. One screening approach is to ask patients to record a 30 second electrocardiogram (ECG, measuring heart activity) multiple times each day for 1–4 weeks. AF can be identified from the ECG as it causes an irregular heart rhythm and causes changes to the ECG signal morphology. This approach has been shown to be acceptable to elderly patients, to identify undiagnosed AF, and to successfully prompt the initiation of anticoagulation treatment [7]. However, the ECG recordings must be manually reviewed to diagnose AF, which is time-consuming and costly.

The use of an automated algorithm to identify potentially abnormal ECGs and exclude the remainder from manual review has been proposed to boost the efficiency of the review process [8]. The algorithm configuration determines the safety and potential benefit of using such an approach: the more sensitive an algorithm is to AF, the safer it is; and the greater its positive predictive value, the fewer ECGs it will send for review. Such an algorithm could reduce the number of recordings sent for review by 88% whilst still ensuring that all patients with AF are correctly identified [8]. Nonetheless, even with this algorithm, approximately 35 ECGs would need to be manually reviewed to find one ECG with AF. Reducing the manual review workload even further would make AF screening more cost-effective, which may result in the greater adoption of AF screening and more frequent screening checks to identify AF earlier [9].

The aim of this study was to evaluate the impact of four different AF screening algorithm configurations on the manual review workload and identification of AF patients. The assessment complements previously reported evidence on the safety of using an AF screening algorithm with handheld ECG devices [8].

## 2 Methods

### 2.1 Dataset

**Figure 1:**
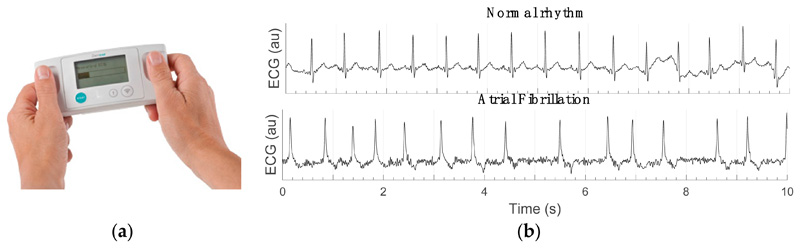
Screening for atrial fibrillation (AF) using a handheld electrocardiogram (ECG) device: (**a**) the Zenicor EKG-2 handheld ECG device was used to record single-lead ECGs; (**b**) ECGs exhibiting AF were identified using an automated algorithm, followed by a ‘first filter’ reviewer to correct any algorithm misclassifications, followed by two experts to provide AF diagnoses (10 s ECG excerpts are shown in arbitrary units—au).

The dataset used in this study was acquired in the SAFER (Screening for Atrial Fibrillation with ECG to Reduce stroke) Feasibility Study (ISRCTN 16939438). Briefly, the SAFER Feasibility Study aimed to assess the feasibility of screening for AF in primary care, and to inform the design of the SAFER Trial (a randomised controlled trial to assess the effectiveness and cost-effectiveness of AF screening). Participants used the Zenicor EKG-2 device (Zenicor Medical Systems AB) shown in Figure 1 to take four 30 s single-lead ECG recordings each day for 1–4 weeks. A total of 2141 participants aged 65 and over took part in the study. They recorded 162,515 ECGs in total, a median (lower–upper quartiles) of 61 (53–111) ECGs per participant.

Participants exhibiting AF were identified as follows. Firstly, all ECG recordings were analysed using the Cardiolund ECG Parser algorithm (Cardiolund AB) [8,10]. This algorithm classified recordings as either low or high signal quality, and as either pathological (abnormal rhythm) or non-pathological (normal sinus rhythm or minor rhythm deviations). Secondly, ‘first filter’ reviews were performed (by a nurse in this study) to correct any algorithm misclassifications and identify the participants requiring further review (who had to have an algorithm classification indicating an abnormal heart rhythm). Thirdly, two expert reviewers (cardiologists in this study) independently reviewed the recordings from these identified participants in order to determine which participants exhibited AF. Any differences between expert reviewer classifications were resolved where possible to reach a final classification for each participant. A participant was classified as having AF if one or more of their recordings exhibited AF for its full 30s duration (or at least all of the readable portions of the recording). A total of 54 participants were found to have AF. Recordings exhibiting AF were labelled on an ad hoc basis by the expert reviewers.

All participants gave informed consent to participate in the study. The study was conducted in accordance with the Declaration of Helsinki and was approved by the London Central NHS Research Ethics Committee (18/LO/2066).

### 2.2 AF Screening Algorithm Configurations

The dataset was used to evaluate four potential AF screening algorithm configurations which determined which classes of ECG recordings are reviewed, as listed in Table 1. The configurations range from reviewing all ECG recordings classified as anything other than high quality and non-pathological (Config. 1), to only recordings classified as pathological (Config. 2), to only recordings with specific pathological classifications that are more indicative of AF (Configs. 3 and 4).

### 2.3 Evaluating the Impact on Manual Review Workload and Identification of AF Patients

The manual review workload associated with each AF screening algorithm configuration was evaluated retrospectively as follows. The number of first filter reviews was calculated as the number of recordings meeting each algorithm configuration’s criteria. The number of expert reviews was calculated as the number of these recordings which still met the criteria after the first filter had corrected any algorithm misclassifications. It was assumed that expert review would be conducted independently by two expert reviewers, as was the case in the SAFER Feasibility Study.

The number of AF patients identified by each AF screening algorithm was calculated as follows. An AF patient was assumed to be identified when using an algorithm configuration if: (i) they were diagnosed with AF in the SAFER Feasibility Study; and (ii) at least one of their ECG recordings which would be sent for expert review under this screening algorithm’s criteria was labelled as AF by one of the expert reviewers.

## 3 Results and Discussion

The results are presented in Table 2.

### 3.1 The Impact of AF Screening Algorithm Configurations on the Manual Review Workload

Config. 1 required 30,165 manual reviews consisting of: 20,155 first filter reviews, and two sets of 5005 expert reviews. The proportion of ECG recordings requiring first filter reviews (12.4%) was similar to the 12.2% reported previously for a similar configuration in the STROKESTOP study (in which short episodes of slow heart rate (HR) were not used, which accounted for 0.2% of ECGs in this study) [8].

Under Config. 2, low quality recordings were excluded from first filter review, reducing the number of first filter reviews considerably by 23%. This strategy had less impact on the number of expert reviews (reduced by 9%) as most low-quality recordings were manually excluded by the first filter prior to expert review in the SAFER Feasibility Study. Overall, the exclusion of low-quality recordings reduced the total number of reviews by 18.6%. In comparison to the STROKESTOP study, the potential benefit of excluding low quality recordings was found to be greater in this study because a greater proportion of recordings were of low quality in this study (2.9% vs. 1.0%) [8].

In Configs. 3 and 4, recordings with certain pathological classifications were excluded. Configs. 3 and 4 reduced the total number of reviews by a further 24.4% and 26.1%, respectively, in comparison to only excluding low quality recordings (Config. 2), and by 38.4% and 40.0%, respectively, in comparison to Config. 1. These configurations substantially reduced the number of both first filter and expert reviews. A similar proportion of recordings was classified as ‘irregular sequence’ in this study (7.6%) as in STROKESTOP (7.2%) [8].

### 3.2 The Impact of Alternative Strategies on Identifying AF Patients

It can be seen from Table 1 that, in this study, Config. 3 was found to require the least number of manual reviews whilst still identifying all 54 AF patients. Whilst Config. 4 would have provided a very slight further reduction in the number of reviews (2%), it would also have missed one AF patient, making Config. 3 most appropriate. The patient missed by Config. 4 was diagnosed with AF based on a recording classified as a fast regular rhythm. This highlights the potential benefit of reviewing recordings classified as either ‘irregular sequence’ or ‘fast regular’ (i.e., Config. 3) to identify all AF patients. Similarly, Config. 4 would have identified most AF patients but not all in the STROKESTOP study (95% of AF patients) [8].

### 3.3 The Importance of the First Filter

In the four screening algorithm configurations, the first filter excluded between 70.4% and 75.2% of ECG recordings prior to expert review. This is beneficial for two reasons. Firstly, it reduces the overall number of manual reviews required if two expert reviewers are used, since every recording excluded by the first filter potentially avoids two additional expert manual reviews. Secondly, this reduces costs as an expert reviewer’s time is likely to be more expensive than that of a first filter. Thus, it appears beneficial to separate the manual review process into these two roles.

### 3.4 Limitations

There are several limitations to this study. First, we assumed that all recordings sent for expert review would be reviewed, whereas in an AF screening programme an expert would likely stop reviewing a patient’s recordings if they found a single recording exhibiting AF. Second, in the SAFER Feasibility Study, all potentially clinically relevant recordings were sent for expert review, rather than only those potentially exhibiting AF. For these two reasons, the numbers of expert reviews presented here are likely to be overestimates of those required in a screening programme. Third, it was assumed that recordings labelled by the algorithm as non-pathological and high quality would not exhibit AF, as it has previously been observed that only approximately one in 11,600 of such recordings exhibit AF [8]. Fourth, we did not assess how different AF screening algorithm configurations would impact the costs of screening—an important consideration when deciding whether to implement AF screening [11]. In this analysis, all manual reviews were treated with the same importance, whereas in reality, expert reviews are likely to be more expensive.

### 3.5 Future Work

This study provides several directions for future research. First, it highlights the importance of automating as much of the manual review process as possible to reduce the workload, whilst ensuring AF patients are reliably identified. Therefore, it would be beneficial to develop the algorithm further to increase its positive predictive value (i.e., the proportion of recordings sent for review which do exhibit AF) whilst maintaining a high sensitivity. This could potentially be achieved by analysing not only the heart rhythm and QRS complexes in ECG recordings, but also P-wave characteristics, mimicking the approach taken by manual reviewers [7]. We have previously reported a P-wave quality index at this Conference which may be useful for such work [12]. Second, it is important to assess the accuracy of manual reviews. This could be achieved by assessing the interobserver variability between reviewers such as between the two expert reviewers in the SAFER Feasibility Study. Third, the dataset presented here provides an ideal opportunity to create a benchmark training dataset of labelled ECG recordings, which could be used in reviewer training. Finally, this study was a retrospective analysis, and future studies should verify the findings prospectively.

### 3.6 Perspectives

Future research on the association between AF burden and stroke risk would help determine whether it is important to identify individuals with only a low number of AF occurrences during screening. If an association was strong enough to indicate that patients with a low AF burden should not receive anticoagulation, then potentially a patient’s recordings could only be sent for review if they exhibited potential AF sufficiently frequently. While many studies support a positive relationship between AF burden and stroke risk, this may be insufficiently strong to outweigh other patient characteristics that are normally taken into account using the CHA2DS2-VASc score to assess stroke risk and guide anticoagulation [13]. Indeed, AF is also a dynamic arrhythmia, and the AF burden assessed at one time point may not necessarily be the same in the next monitoring period. Furthermore, multivariate analyses have shown that patients’ clinical characteristics, and not AF pattern, independently increase stroke risk. Therefore, based on existing evidence, it is of paramount importance that as many cases of AF as possible are diagnosed, regardless of AF burden, necessitating high specificity in a screening algorithm. Previous validation of the low false-negative rate of the current algorithm provides confidence that this can be achieved [8].

## 4 Conclusions

This study independently verified the manual review workload associated with using handheld ECG devices for AF screening. This highlighted the importance of an automated algorithm in identifying potentially pathological ECG recordings for review, and vastly reducing the number of manual reviews required. In addition, this study indicates that the workload could be reduced further by using a screening algorithm configuration which only identifies certain pathological recordings for review, whilst still identifying AF patients accurately. The assessment of the proposed algorithm configurations in future prospective studies is crucial to verify their safety and associated workload.

## Figures and Tables

**Table 1:** AF screening algorithm configurations assessed: the classes of ECG recordings identified for review by each algorithm configuration.

AF Screening Algorithm Configuration	Pathological Recordings^1^			Low Quality Recordings
	Irregular Sequence	Fast Regular	Other	
Config. 1: All pathological or low quality	✓	✓	✓	✓
Config. 2: All pathological	✓	✓	✓	
Config. 3: Selected pathological	✓	✓		
Config. 4: Only irregular sequences	✓			

1 Pathological classifications were: (i) irregular sequence—irregularly irregular rhythm for entire 30 s recording; (ii) fast regular—fast heart rate (HR) of ≥120 beats per minute (bpm); (iii) other (slow heart rate of ≤45 bpm for entire recording, short episode of slow HR, short episode of fast HR, ≥5 ventricular extra systoles, or a pause of >2.2 s or skipped QRS complex).

**Table 2:** The number of manual reviews required and the number of AF patients identified when using each manual review strategy.

AF Screening Algorithm Configuration	No. of Manual Reviews			No. of AF Patients Identified
	First Filter	Expert	Total	
Config. 1: All pathological or low quality	20,155	5005 × 2	30,165	54
Config. 2: All pathological	15,421	4570 × 2	24,561	54
Config. 3: Selected pathological	11,975	3299 × 2	18,573	54
Config. 4: Only irregular sequences	11,748	3198 × 2	18,144	53

## References

[1] Adderley NN, Ryan R, Nirantharakumar K, Marshall T (2019). Prevalence and treatment of atrial fibrillation in UK general practice from 2000 to 2016. Heart.

[2] Wolf PA, Abbot RD, Kannel WB (1991). Atrial fibrillation as an independent risk facor for stroke: the Framingham study. Stroke.

[3] Perera KK, Vanassche T, Bosch J, Swaminathan B, Mundl H, Giruparajah M, Barboza MM, O’Donnell MM, Gomez-Schneider M, Hankey GG (2016). Global Survey of the Frequency of Atrial Fibrillation-Associated Stroke. Stroke.

[4] Hart RR, Pearce LL, Aguilar MI (2007). Meta-analysis: antithrombotic therapy to prevent stroke in patients who have nonvalvular atrial fibrillation. Ann Intern Med.

[5] Public Health England (2015). Atrial Fibrillation Prevalence Estimates in England: Application of Recent Population Estimates of AF in Sweden.

[6] Krijthe BB, Kunst A, Benjamin EE, Lip GYH, Franco OO, Hofman A, Witteman JCM, Stricker BB, Heeringa J (2013). Projections on the number of individuals with atrial fibrillation in the European Union, from 2000 to 2060. Eur Heart J.

[7] Svennberg E, Engdahl J, Al-Khalili F, Friberg L, Frykman V, Rosenqvist M (2015). Mass screening for untreated atrial fibrillation: the STROKESTOP study. Circulation.

[8] Svennberg E, Stridh M, Engdahl J, Al-Khalili F, Friberg L, Frykman V, Rosenqvist M (2017). Safe automatic one-lead electrocardiogram analysis in screening for atrial fibrillation. Europace.

[9] Aronsson M, Svennberg E, Rosenqvist M, Engdahl J, Al-Khalili F, Friberg L, Frykman V, Levin LÅ (2017). Designing an optimal screening program for unknown atrial fibrillation: A cost-effectiveness analysis. Europace.

[10] Stridh M, Rosenqvist M Automatic Screening of Atrial Fibrillation in Thumb-ECG Recordings.

[11] Jones NN, Taylor CC, Hobbs FDR, Bowman L, Casadei B (2020). Screening for atrial fibrillation: A call for evidence. Eur Heart J.

[12] Tecelão D, Charlton P (2019). Automated P-Wave Quality Assessment for Wearable Sensors. Proceedings.

[13] Chen LL, Chung MM, Allen LL, Ezekowitz M, Furie KK, McCabe P, Noseworthy PP, Perez MV, Turakhia MP (2018). Atrial Fibrillation Burden: Moving Beyond Atrial Fibrillation as a Binary Entity: A Scientific Statement From the American Heart Association. Circulation.

